# “HLA-G 3′UTR gene polymorphisms and rheumatic heart disease: a familial study among South Indian population”

**DOI:** 10.1186/s12969-017-0140-x

**Published:** 2017-02-01

**Authors:** Maheshkumar Poomarimuthu, Sivakumar Elango, Sambath Soundrapandian, Jayalakshmi Mariakuttikan

**Affiliations:** 1 Department of Immunology, School of Biological Sciences, Madurai Kamaraj University, Madurai, 625 021 Tamil Nadu India; 2 Institute of Child Health and Research Centre, Government Rajaji Hospital, Madurai, Tamil Nadu India

**Keywords:** Human leukocyte antigen G, Rheumatic fever, Rheumatic heart disease, Polymorphism, Autoimmunity

## Abstract

**Background:**

Rheumatic heart disease (RHD) is an autoimmune disease where cross reactive CD4^+^ T cells are involved in the pathogenesis of valvular damage. Human Leukocyte Antigen-G (HLA-G), an immunosuppressive molecule playing a crucial role in the inhibition of T cell response is associated with the pathogenesis of various autoimmune and inflammatory diseases. Genetic polymorphisms within the 3′untranslated region (UTR) of HLA-G influences its expression and thus disease pathogenesis. Hence, the present study aims to unravel the association of 14 bp Ins/Del (rs66554220) and +3142 C*/*G (rs1063320) polymorphisms in 3′ UTR of HLA-G with RHD.

**Methods:**

This familial study consists of 99 RHD families (99 RHD patients, 140 parents and 126 healthy siblings). The 14 bp Ins/Del and +3142 C*/*G polymorphisms were evaluated by PCR using sequence specific primers and its transmission disequilibrium (TD) was tested by TD test in 70 trio families.

**Results:**

The frequency of +3142 C/C genotype was high in patients with combined valvular lesions (CVL) (OR = 5.88; p_c_ = 0.012) and pooled RHD patients (P: OR = 2.76; *p* = 0.043; p_c_ = 0.076) when compared to healthy siblings. Under the additive (OR = 5.50; p_c_ = 0.026) and recessive genetic model (OR = 5.88; p_c_ = 0.012), the +3142 C/C genotype was significantly associated with CVL in patients.

**Conclusion:**

The results suggest that the +3142 C/C genotype may be associated with minor risk for the development of RHD and is more likely to influence the severity of the disease.

**Electronic supplementary material:**

The online version of this article (doi:10.1186/s12969-017-0140-x) contains supplementary material, which is available to authorized users.

## Background

RHD is an autoimmune disease occurring as a result of group A β-hemolytic Streptococcus (GAS) infection followed by rheumatic fever (RF) [1]. Molecular mimicry between streptococcal M protein and human cardiac proteins leads to the development of RHD in a susceptible host [2]. RHD causes major cardiac illness, subsequently leading to high morbidity and mortality among children in developing countries and also in certain indigenous populations within developed countries. Globally, over 15 million cases of RHD have been reported and its prevalence in India ranges from 2–2.5 million [3, 4]. It is an inflammatory condition resulting in chronic valvular damage specifically mediated by cellular immune response. The aberrant immune response against host cardiac proteins may account for the progressive valvular damage [5–9].

HLA-G is a non-classical class-I HLA molecule associated with anti-inflammatory and immuno-modulatory properties [10, 11] which interacts with inhibitory receptors (ILT2/ILT4/KIR2DL4) present on various immune cells. It inhibits proliferation of B cells, T cells and natural killer cells; and also induces regulatory T cells (T-reg) [12–15]. The polymorphic 3’UTR of HLA-G plays a vital role in the regulation of HLA-G expression. The 14bp Ins/Del polymorphism regulates HLA-G expression by influencing the splicing pattern and stability of mRNA. Whereas, the +3142 C*/*G polymorphism enhances the affinity for micro RNAs and downregulate the expression of HLA-G [16–19]. Thus, both the polymorphisms influence HLA-G expression and are associated with various autoimmune and inflammatory diseases [20, 21]. Several studies have demonstrated the genetic predisposition to RHD, especially those genes which are involved in immune response [22]. However, the role of HLA-G in the pathogenesis of RHD has not been reported yet. Hence, the aim of this study is to evaluate the possible role of HLA-G gene polymorphisms with particular importance to 14bp Ins/Del (rs66554220) and +3142C/G (rs1063320) variants towards the development of RHD in South Indian population.

## Methods

### Study population

This family based retrospective study consisted of 365 individuals (99 RHD patients, 140 parents and 126 healthy siblings, which includes 70 trios), recruited from the Institute of Child Health and Research Centre, Government Rajaji Hospital, Madurai, India. The study was approved by ethics committees of the participating institutions and was carried out in accordance with the declaration of Helsinki. Patients with ARF were diagnosed based on modified Jones criteria. They clinically present with migrating polyarthritis, carditis and chorea. Based on echocardiographic data, the patients were classified into three subgroups: (i) rheumatic fever (RF) - patients without residual valvular disease; (ii) mitral valvular lesions (MVL) - patients with mitral valvular lesions only; (iii) combined valvular lesions (CVL) - patients with multiple valvular lesions along with MVL. Further, the severity of valve damage was assessed by investigations like echo cardiogram showing Mitral/Aortic/Tricuspid valve morphology, color flow doppler MR/AR/TR jet, vena contracta width, effective regurgitant orifice area, left atrium (LA) and left ventricle (LV) size, pulmonary arterial pressure, pulmonary vein flow and hepatic vein flow. In addition, regurgitaion is clinically diagnosed by prominent pulsations of the jugular veins, systolic pulsations of the liver and a blowing holosystolic murmur at the lower left sternal border which increases in intensity during inspiration and expiration for TR and MR respectively. Currently, all the patients in the study group are in quiescent phase, under penicillin (tablet) prophylaxis against rheumatic fever recurrence. Patients with carditis were treated with steroids (oral prednisone 2mg/Kg) initially and subsequently treated with aspirin tablets. Antifailure drugs like frusemide, digoxin and enalapril were given appropriately to the patients with congestive cardiac failure. None of the patients underwent surgery for valvular heart disease in this study group. Siblings, free from autoimmune and cardiac diseases were considered as controls. Like the patients, the siblings are under follow-up at regular intervals. None of the siblings developed any symptoms of ARF like, polyarthritis, carditis, chorea, erythema marginatum and subcutaneous nodules during the follow-up period. The siblings are advised to be under regular follow-up. Blood samples (2.5 ml) were collected from all the participating individuals with their consent. For children (<18 years), the consent was duly signed by their parents.

### Genotyping of HLA-G gene polymorphisms

Genomic DNA was extracted from the blood samples by salting out method [23]. HLA-G 14 bp Ins/Del and +3142 C/G polymorphisms were examined by direct PCR DNA amplification method [24]. Further, validation of 14 bp Ins/Del polymorphism was also performed by PCR-SSP [25].

### Statistical analysis

Hardy-Weinberg equilibrium (HWE), TDT, linkage disequilibrium (LD) and haplotype analysis was performed using *Haploview v4.2* [26]*.* The HLA-G allelic and genotypic frequencies of patients and siblings were compared using the Fisher’s exact test or Pearson’s *χ* ^2^ test with Yates correction using *Epi Info v*7. The odds ratio (OR) with 95% confidence interval (CI) was calculated and the p <0.05 was considered statistically significant.

## Results

### Characteristics of the study population

This familial study included 99 RHD patients (52 males, 47 females, mean age 11.2 ± 2.5 years), 126 healthy siblings (75 males and 51 females, mean age 12.2 ± 4.3 years) and 140 parents (70 mothers and 70 fathers). The demographic and clinical characteristics of the study subjects is represented in Table 1. In this study group, migrating polyarthritis was present in 80 patients and carditis was seen in 56 patients. In addition, chorea was present in 7 patients while erythema marginatum and subcutaneous nodules were not seen even in a single patient. Out of 99 patients, 10 (10.1%) patients had RF, 67 (67.7%) had MVL and 22 (22.2%) had CVL.

**Table 1:** Demographic characteristics and clinical details of patients and healthy siblings

Parameters		Patients *N* = 99 (%)	Healthy siblings *N* = 126 (%)
Age, in years (mean ± SD)		11.2 ± 2.5	12.2 ± 4.3
Gender			
Male		52 (52.5)	75 (59.5)
Female		47 (47.5)	51 (40.5)
Patient classification			
Rheumatic Fever		10 (10.1)	
Valvular Lesions			
MVL		67 (67.7)	
Mitral Regurgitation	Mild	24 (35.8)	
	Moderate - Severe	35 (52.2)	
*MR with MS*		3 (4.5)	
Mild MR, Mild MS		1 (33.3)	
Moderate MR, Mild MS		2 (66.7)	
*MR with chorea*		5 (7.5)	
Mild MR with chorea		4 (80.0)	
Moderate MR with chorea		1 (20.0)	
CVL		22 (22.2)	
*MR, AR*		17 (77.3)	
Mild MR, Mild AR		6 (35.3)	
Moderate MR, Mild AR		6 (35.3)	
Moderate MR, Mod AR		1 (5.9)	
Severe MR, Mild AR		3 (17.6)	
Severe MR, Mod AR		1 (5.9)	
*MR, TR*		2 (9.1)	
Severe MR, Mild TR		2 (100)	
*MR, MS, AR*		3 (13.6)	
Mild MR, Severe MS, Mild AR		1 (33.3)	
Mod MR, Mod MS, Mild AR		2 (66.7)	

*SD* Standard Deviation, *MVL* Mitral Valvular Lesions, *MR* Mitral Regurgitation, *MS* Mitral Stenosis, *CVL* Combined Valvular Lesions, *AR* ortic Regurgitaion, *TR* Triscuspid Regurgitation, *Mod* Moderate

### Transmission disequilibrium test (TDT)

In the present study, 70 trio families were included for TDT analysis (Table 2). No Mendelian error was observed and the families were in HWE expectations. In single marker analysis, Del allele (32 T vs 28 UT; *p* = 0.606) and G allele (27 T vs 23 UT; *p* = 0.572) were overtransmitted, however no statistical significance was observed. Likewise, the haplotype analysis also showed no significance among Ins/G (28 T vs. 31 UT; *p* = 0.697), Del/G (28.5 T vs. 20.5 UT; *p* = 0.255) and Del/C haplotypes (23.5 T vs. 27.5 UT; *p* = 0.578).

**Table 2:** TDT of HLA-G polymorphisms in 70 trio families

	T	UT	*χ* ^2^	*p* value
14 bp Ins/Del				
Del allele	32	28	0.267	0.6056
+3142 C/G				
G allele	27	23	0.320	0.5716
Haplotypes				
Ins/G	28	31	0.152	0.6969
Del/G	28.5	20.5	1.295	0.2552
Del/C	23.5	27.5	0.309	0.5782

*TDT* transmission disequilibrium test, *T* transmitted, *UT* untransmitted

### Linkage disequilibrium analysis

Pairwise linkage disequilibrium analysis was carried out for the two polymorphic sites of the HLA-G gene in the study population. The haplotype analysis predicted a single block of high LD in healthy siblings (D’ = 1, LOD = 10.57 and r^2^ = 0.38), RHD patients (D’ = 1, LOD = 10.56 and r^2^ = 0.32) and Trios (D’ = 0.966; LOD = 17.82; r^2^ = 0.285) between HLA-G 14 bp Ins/Del and +3142 C/G polymorphism (Additional file 1: Figure S1).

### Genotype and Allele distribution in RHD patients and healthy siblings

The genotype and allele frequencies of 14bp Ins/Del and +3142 C/G polymorphisms for pooled (P) RHD patients and healthy siblings were shown in Table 3. The data was further stratified based on gender (male (M) and female (F)). There were no significant differences observed for the genotype and allele frequencies of these two polymorphisms between RHD patients and healthy siblings.

**Table 3:** HLA-G genotype, allele and haplotype frequencies in patients and healthy siblings

	Pooled				Male				Female			
	Patients (99) n (%)	Healthysiblings (126) n (%)	OR (95% CI)	p_c_	Patients (52) n (%)	Healthy siblings (75) n (%)	OR (95% CI)	p_c_	Patients (47) n (%)	Healthy siblings (51) n (%)	OR (95% CI)	p_c_
14 bp Ins/Del												
Genotypes												
Ins/Ins	16 (16.2)	27 (21.4)	0.71 (0.36-1.40)	0.4084	9 (17.3)	24 (32.0)	0.44 (0.18-1.04)	0.0987	7 (14.9)	3 (5.9)	2.86 (0.69-11.77)	0.2418
Del/Del	35 (35.3)	36 (28.6)	1.37 (0.78-2.40)	0.3128	18 (34.6)	19 (25.3)	1.53 (0.71-3.32)	0.3755	17 (36.2)	17 (33.3)	1.17 (0.51-2.68)	0.8791
Ins/Del	48 (48.5)	63 (50.0)	0.94 (0.55-1.59)	0.9272	25 (48.1)	32 (42.7)	1.24 (0.61-2.53)	0.6734	23 (48.9)	31 (60.8)	0.62 (0.28-1.38)	0.3296
Alleles												
Ins	80 (40.4)	117 (46.4)	0.78 (0.54-1.14)	0.2368	43 (41.3)	80 (53.3)	0.62 (0.37-1.02)	0.0797	37 (39.4)	37 (36.3)	1.14 (0.64-2.03)	0.7657
Del	118 (59.6)	135 (53.6)	1.28 (0.88-1.86)	0.2368	61 (58.7)	70 (46.7)	1.62 (0.98-2.70)	0.0797	57 (60.6)	65 (63.7)	0.88 (0.49-1.56)	0.7657
+3142 C/G												
Genotypes												
C/C	12 (12.1)	6 (4.8)	2.76 (0.99-7.63)	0.0763^a^	7 (13.5)	3 (4.0)	3.68 (0.90-14.98)	0.1121	5 (10.6)	3 (5.9)	1.94 (0.44-8.62)	0.6041
G/G	48 (48.5)	66 (52.4)	0.86 (0.50-1.45)	0.6556	27 (51.9)	44 (58.7)	0.76 (0.37-1.55)	0.5680	21 (44.7)	22 (43.1)	1.06 (0.48-2.37)	0.9602
C/G	39 (39.4)	54 (42.8)	0.87 (0.51-1.48)	0.6985	18 (34.6)	28 (37.3)	0.87 (0.41-1.82)	0.8556	21 (44.7)	26 (51.0)	0.81 (0.37-1.78)	0.7431
Alleles												
C	63 (31.8)	66 (26.2)	1.31 (0.87-1.98)	0.2280	32 (30.8)	34 (22.7)	1.52 (0.86-2.66)	0.1927	31 (33.0)	32 (31.4)	1.08 (0.59-1.96)	0.9302
G	135 (68.2)	186 (73.8)	0.76 (0.50-1.15)	0.2280	72 (69.2)	116 (77.3)	0.66 (0.37-1.16)	0.1927	63 (67.0)	70 (68.6)	0.93 (0.51-1.69)	0.9302
Haplotypes												
Ins/G	80 (40.4)	117 (46.4)	0.78 (0.54-1.40)	0.2368	43 (41.3)	80 (53.3)	0.62 (0.37-1.02)	0.0797	37 (39.4)	37 (36.3)	1.14 (0.64-2.03)	0.7657
Del/G	55 (27.8)	69 (27.4)	1.02 (0.67-1.55)	0.9898	29 (27.9)	36 (24.0)	1.22 (0.69-2.16)	0.5813	26 (27.6)	33 (32.3)	0.80 (0.43-1.48)	0.5756
Del/C	63 (31.8)	66 (26.2)	1.31 (0.87-1.98)	0.2280	32 (30.8)	34 (22.7)	1.49 (0.85-2.62)	0.2148	31 (33.0)	32 (31.4)	1.11 (0.61-2.01)	0.8567

OR-odds ratio, CI-confidence interval, Pc, Yates corrected p value

a - *χ* ^2^ = 6.3692, uncorrected *p* = 0.0434

In 14 bp Ins/Del polymorphism, an increased frequencies of Ins/Ins genotype (OR = 2.86; p_c_ = 0.241) and Ins allele (OR = 1.14; p_c_ = 0.766) were observed in female RHD patients. Moderately increased frequencies were noticed in Del/Del genotype (P: OR = 1.37; p_c_ = 0.312) and Del allele (P: OR = 1.28; p_c_ = 0.236) among pooled RHD patients. The subgroup analysis showed increased frequencies of Del/Del genotype in CVL (OR = 1.73; p_c_ = 0.363) and MVL patients (OR = 1.39; p_c_ = 0.383) (Table 4). In addition, increased frequency of Del allele was observed in MVL (OR = 1.37; pc = 0.184) and CVL patients (OR = 1.25; 0.607).

**Table 4:** HLA-G genotype, allele and haplotype frequencies in patient sub groups

	Healthy Siblings (126) n (%)	RF 10 n (%)	OR (95% CI)	p_c_	MVL 67 n (%)	OR (95% CI)	p_c_	CVL 22 n (%)	OR (95% CI)	p_c_
14 bp Ins/Del										
Genotypes										
Ins/Ins	27 (21.4)	2 (20.0)	0.92 (0.18-4.57)	0.7680	9 (13.4)	0.57 (0.25-1.29)	0.2446	5 (22.7)	1.08 (0.36-3.19)	0.8854
Del/Del	36 (28.6)	2 (20.0)	0.62 (0.13-3.09)	0.8294	24 (35.8)	1.39 (0.74-2.62)	0.3829	9 (40.9)	1.73 (0.68-4.40)	0.3630
Ins/Del	63 (50.0)	6 (60.0)	1.5 (0.40-5.57)	0.7792	34 (50.8)	1.03 (0.57-1.86)	0.9581	8 (36.4)	0.57 (0.22-1.46)	0.3421
Alleles										
Ins	117 (46.4)	10 (50.0)	1.15 (0.46-2.86)	0.9399	52 (38.8)	0.73 (0.48-1.12)	0.1837	18 (40.9)	0.80 (0.42-1.53)	0.6070
Del	135 (53.6)	10 (50.0)	0.87 (0.35-2.15)	0.9399	82 (61.2)	1.37 (0.89- 2.09)	0.1837	26 (59.1)	1.25 (0.65-2.40)	0.6070
+3142 C/G										
Genotypes										
C/C	6 (4.8)	0	0	0.9250	7 (10.5)	2.33 (0.75-7.25)	0.2306	5 (22.7)	5.88 (1.62-21.39)	**0.0116** ^**a**^
G/G	66 (52.4)	4 (40.0)	0.61 (0.16-2.25)	0.6705	34 (50.7)	0.94 (0.52-1.69)	0.9481	10 (45.5)	0.76 (0.30-1.89)	0.7124
C/G	54 (42.8)	6 (60.0)	2.0 (0.54-7.44)	0.4714	26 (38.8)	0.84 (0.46-1.55)	0.6962	7 (31.8)	0.62 (0.24-1.63)	0.4618
Alleles										
C	66 (26.2)	6 (30.0)	1.21 (0.44-3.27)	0.9136	40 (29.9)	1.20 (0.75-1.90)	0.5174	17 (38.6)	1.78 (0.91-3.46)	0.1300
G	186 (73.8)	14 (70.0)	0.83 (0.30-2.24)	0.9136	94 (70.1)	0.83 (0.52-1.33)	0.5174	27 (61.4)	0.56 (0.29-1.10)	0.1300
Haplotypes										
Ins/G	117 (46.4)	10 (50.0)	1.15 (0.46-2.86)	0.9399	52 (38.8)	0.73 (0.48-1.12)	0.1837	18 (40.9)	0.80 (0.42-1.53)	0.6070
Del/G	69 (27.4)	4 (20.0)	0.66 (0.21-2.05)	0.6491	42 (31.3)	1.21 (0.76-1.91)	0.4835	9 (20.5)	0.68 (0.31-1.49)	0.4372
Del/C	66 (26.2)	6 (30.0)	1.21 (0.44-3.27)	0.9136	40 (29.9)	1.19 (0.75-1.91)	0.5174	17 (38.6)	1.78 (0.91-3.46)	0.1300

RF- Rheumatic Fever, MVL-Mitral Valvular Lesions, CVL-Combined Valvular Lesions, CI-confidence interval, OR-odds ratio, p_c_-Yates corrected p value

a - *χ* ^2^ = 6.3692, Fisher Exact P (P_f_) = 0.0117

Bold - significant p value (<0.05)

The +3142 C/C genotype frequency was relatively high in pooled RHD patients (P: OR = 2.76; *p* = 0.043; p_c_ = 0.076). Significantly increased frequency of +3142 C/C genotype was found in CVL patients (OR = 5.88; p_c_ = 0.012) when compared to healthy siblings (Table 4). In the genetic model analysis, the additive (OR = 5.50; p_c_ = 0.026) and recessive pattern (OR = 5.88; p_c_ = 0.012) of +3142 C/C genotype was inherited significantly among CVL patients (Table 5) when compared to healthy siblings. The +3142 C/C genotype frequency was higher in male RHD patients (M: OR = 3.68; p_c_ = 0.112) when compared to female RHD patients (F: OR = 1.94; p_c_ = 0.604).

**Table 5:** Analysis of Genotype as risk factor in CVL patients

Study models	OR (95% CI)	p_c_
CC + CG vs GG^a^ CC vs GG^b^ CC vs GG + CG^c^ CG vs CC + GG^d^	1.32 (0.53-3.28) 5.50 (1.41-21.44) 5.88 (1.62-21.39) 0.62 (0.24-1.63)	0.7124 **0.0261** **0.0116** 0.4618

*CVL* Combined Valvular Lesions, CI-confidence interval, *OR* odds ratio, p_c_-Yates corrected p value. Bold - significant p value (<0.05)

a-Dominant Effect

b-Additive Effect

c- Recessive Effect

d-Co-dominant Effect

The three possible haplotypes observed in the present study were Ins/G, Del/G and Del/C. The frequency distribution of these haplotypes among the study groups did not show statistical significance (Table 3). However, increased frequency of Del/C haplotype was observed in pooled RHD (P: OR = 1.31; p_c_ = 0.228) and CVL patients (OR = 1.78; p_c_ = 0.130) (Table 4) while Ins/G haplotype was found to be high among female RHD patients (F: OR = 1.14; p_c_ = 0.766).

## Discussion

RHD is an autoimmune disease characterised by persistent inflammatory reaction towards valvular tissue. HLA-G has an immunoregulatory function and could play a vital role in the pathogenesis of immune-mediated diseases, including RHD. To our knowledge, this is the first study to investigate the association of HLA-G 14 bp Ins/Del and +3142 C/G polymorphisms with the pathogenesis of RHD. Hence, we validate our findings with previous reports on various autoimmune diseases such as systemic lupus erythematosus (SLE), rheumatoid arthritis (RA), type 1 diabetes (T1D), idiopathic dilated cardiomyopathy (IDC), and juvenile idiopathic arthritis (JIA) [27–38].

In the present study, the TDT analysis showed that the Del allele was overtransmitted in RHD patients. In addition, the Del/Del genotype and Del allele were overrepresented in pooled RHD patients when compared to healthy siblings. This is in agreement with the previous studies carried out on autoimmune diseases such as T1D, IDC and JIA [33–35]. Increased frequencies of Ins/Ins genotype and Ins allele were observed in female RHD patients, which differ from the previous finding of increased frequency of Del allele among female JIA patients [35]. Increased frequencies of Ins allele and Ins/Ins genotypes were documented in SLE patients [27, 28, 36, 37], however no association of HLA-G 14 bp Ins/Del polymorphism was reported in SLE [31, 38] and RA patients [32, 35].

In this study, the TDT analysis revealed that the +3142 G allele was overtransmitted from parents to RHD patients. We could observe a borderline association of +3142 C/C genotype with susceptibility to RHD. In addition, the frequency of +3142 C/C genotype was significantly high among CVL patients when compared to healthy siblings. Thus the recessive and additive model analysis revealed that +3142 C/C genotype was significantly associated with risk for CVL. Likewise, the +3142 C allele was associated with risk for clinical subtypes of SLE patients in Japanese population [27]. In contrast, the +3142 G/G genotype and G allele were associated with susceptibility to SLE [28, 29] and RA [30] in Brazilian populations. However, no association of +3142 C/G polymorphism was observed in Brazilian and South Indian RA patients [31, 32].

Our results did not reveal any significant difference in the haplotype frequencies of Ins/G, Del/G and Del/C in the study population. However, increased frequencies of Del/C haplotype was observed in pooled RHD patients and among the patient subgroups. Conversely a previous study reported the association of Del/C haplotype with protection to SLE [29] and RA [30]. Several studies reported that Del/C haplotype is associated with high level of HLA-G expression while Ins/G haplotype is associated with low level of HLA-G expression [39]. In addition, there might be other polymorphic loci in the 3’UTR and promoter region with strong LD which could regulate the HLA-G expression [40, 41] and play a conspicuous role in the development and progression of RHD. Several studies showed contradictory results regarding the influence of HLA-G 3’UTR polymorphisms on HLA-G expression and pathogenesis of various autoimmune and inflammatory diseases [27–38, 42–49]. Thus it is hard to speculate the association of HLA-G with the pathogenesis of RHD. The limitation of the present study in ascertaining the definite role of HLA-G in RHD could be due to the lack of soluble HLA-G quantification, thereby causing difficulty to correlate the influence of 3’UTR polymorphisms on HLA-G expression.

## Conclusion

In conclusion, in this pilot study we analysed for the first time the association of HLA G 3’prime UTR polymorphism in the development of RHD and the results suggest that the +3142 C/C genotype may influence the development of RHD and subsequent severity of the disease. While, the 14bp Ins/Del polymorphism is not associated with RHD risk in South Indian population. However, further investigation by increasing the sample cohort with diverse populations are indispensable to elucidate the significance of HLA-G in the etiopathogenesis of RHD.

## Additional file

Additional file 1: Figure S1. Pairwise linkage disequilibrium based on 2 HLA-G Polymorphisms using *HaploView 4.2*. Red squares represent high pair-wise linkage disequilibrium. The numbers in the individual square are D' multiplied by 100. A. Healthy siblings B. RHD patients C. Trio families. (DOC 301 kb)

## Abbreviations

CI: Confidence intervals
CVL: Combined Valvular Lesions
GAS: Group A β-hemolytic Streptococcus
HLA-G: Human Leukocyte Antigen-G
HWE: Hardy-Weinberg equilibrium
IDC: Idiopathic dilated cardiomyopathy
JIA: Juvenile idiopathic arthritis
LD: Linkage disequilibrium
MVL: Mitral valvular lesions
OR: Odds ratio
RA: Rheumatoid arthritis
RF: Rheumatic fever
RHD: Rheumatic heart disease
SLE: Systemic lupus erythematosus
T1D: Type 1 diabetes
TDT: Transmission disequilibrium test
T-reg: Regulatory T cells
UTR: Untranslated region

## References

[1] Guilherme L, Fae K, Oshiro SE, Kalil J (2005). Molecular pathogenesis of rheumatic fever and rheumatic heart disease. Expert Rev Mol Med.

[2] Guilherme L, Kalil J, Cunningham M (2006). Molecular mimicry in the autoimmune pathogenesis of rheumatic heart disease. Autoimmunity.

[3] Seckeler MD, Hoke TR (2011). The worldwide epidemiology of acute rheumatic fever and rheumatic heart disease. Clin Epidemiol.

[4] Kumar RK, Tandon R (2013). Rheumatic fever & rheumatic heart disease: The last 50 years. Indian J Med Res.

[5] Kemeny E, Grieve T, Marcus R, Sareli P, Zabriskie JB (1989). Identification of mononuclear cells and T cell subsets in rheumatic valvulitis. Clin Immunol Immunopathol.

[6] Guilherme L, Cunha-Neto E, Coelho V, Snitcowsky R, Pomerantzeff PM, Assis RV (1995). Human heart-infiltrating T cell clones from rheumatic heart disease patients recognize both streptococcal and cardiac proteins. Circulation.

[7] Guilherme L, Oshiro SE, Fae KC, Cunha-Neto E, Renesto G, Goldberg AC (2001). T cell reactivity against streptococcal antigens in the periphery mirrors reactivity of heart-infiltrating T lymphocytes in rheumatic heart disease patients. Infect Immun.

[8] Bas HD, Baser K, Yavuz E, Bolayir HA, Yaman B, Unlu S (2014). A shift in the balance of regulatory T and T helper 17 cells in rheumatic heart disease. J Investig Med.

[9] Mukhopadhyay S, Varma S, Gade S, Yusuf J, Trehan V, Tyagi S (2013). Regulatory T-cell deficiency in rheumatic heart disease: a preliminary observational study. J Heart Valve Dis.

[10] Carosella ED, Dausset J, Rouas-Freiss N (1999). Immunotolerant functions of HLA-G. Cell Mol Life Sci.

[11] Carosella ED, Rouas-Freiss N, Roux DT, Moreau P, LeMaoult J (2015). HLA-G: An Immune Checkpoint Molecule. Adv Immunol.

[12] Le Gal FA, Riteau B, Sedlik C, Khalil-Daher I, Menier C, Dausset J (1999). HLA-G-mediated inhibition of antigen-specific cytotoxic T lymphocytes. Int Immunol.

[13] Bainbridge DR, Ellis SA, Sargent IL (2000). HLA-G suppresses proliferation of CD4(+) T-Lymphocytes. J Reprod Immunol.

[14] Kapasi K, Albert SE, Yie S, Zavazava N, Librach CL (2000). HLAG has a concentration-dependent effect on the generation of an allo-CTL response. Immunology.

[15] Khalil-Daher I, Riteau B, Menier C, Sedlik C, Paul P, Dausset J (1999). Role of HLA-G versus HLA-E on NK function: HLA-G is able to inhibit NK cytolysis by itself. J Reprod Immunol.

[16] Rousseau P, Le Discorde M, Mouillot G, Marcou C, Carosella ED, Moreau P (2003). The 14 bp deletion-insertion polymorphism in the 3’ UT region of the HLA-G gene influences HLA-G mRNA stability. Hum Immunol.

[17] Rebmann V, van der Ven K, Passler M, Pfeiffer K, Krebs D, Grosse-Wilde H (2001). Association of soluble HLA-G plasma levels with HLA-G alleles. Tissue Antigens.

[18] Castelli EC, Moreau P, Oya e Chiromatzo A, Mendes-Junior CT, Veiga-Castelli LC, Yaghi L (2009). In silico analysis of microRNAS targeting the HLA-G 3’ untranslated region alleles and haplotypes. Hum Immunol.

[19] Hviid TV, Hylenius S, Rorbye C, Nielsen LG (2003). HLA-G allelic variants are associated with differences in the HLA-G mRNA isoform profile and HLA-G mRNA levels. Immunogenetics.

[20] Larsen MH, Hviid TV (2009). Human leukocyte antigen-G polymorphism in relation to expression, function and disease. Hum Immunol.

[21] Rizzo R, Bortolotti D, Bolzani S, Fainardi E (2014). HLA-G Molecules in Autoimmune Diseases and Infections. Front Immunol.

[22] Martin WJ, Steer AC, Smeesters PR, Keeble J, Inouye M, Carapetis J (2015). Post-infectious group A streptococcal autoimmune syndromes and the heart. Autoimmun Rev.

[23] Miller SA, Dykes DD, Polesky HF (1988). A simple salting out procedure for extracting DNA from human nucleated cells. Nucleic Acid Res.

[24] Silva ID, Muniz YC, Sousa MC, Silva KR, Castelli EC, Filho JC (2013). HLA-G 3’UTR polymorphisms in high grade and invasive cervico-vaginal cancer. Hum Immunol.

[25] Hviid TV, Hylenius S, Hoegh AM, Kruse C, Christiansen OB (2002). HLA-G polymorphisms in couples with recurrent spontaneous abortions. Tissue Antigens.

[26] Barrett JC, Fry B, Maller J, Daly MJ (2005). Haploview: analysis and visualization of LD and haplotype maps. Bioinformatics.

[27] Hachiya Y, Kawasaki A, Oka S, Kondo Y, Ito S, Matsumoto I (2016). Association of HLA*-*G 3’ Untranslated Region Polymorphisms with Systemic Lupus Erythematosus in a Japanese Population: A Case–control Association Study. PLoS One.

[28] Lucena-Silva N, de Souza VS, Gomes RG, Fantinatti A, Muniz YC, de Albuquerque RS (2013). HLA-G 3′ untranslated region polymorphisms are associated with systemic lupus erythematosus in 2 Brazilian populations. J Rheumatol.

[29] Consiglio CR, Veit TD, Monticielo OA, Mucenic T, Xavier RM, Brenol JC (2011). Association of the HLA-G gene +3142C > G polymorphism with systemic lupus erythematosus. Tissue Antigens.

[30] Veit TD, de Lima CP, Cavalheiro LC, Callegari-Jacques SM, Brenol CV, Brenol JC (2014). HLA-G +3142 polymorphism as a susceptibility marker in two rheumatoid arthritis populations in Brazil. Tissue Antigens.

[31] Catamo E, Addobbati C, Segat L, Sotero Fragoso T, Domingues Barbosa A, Tavares Dantas A (2014). HLA-G gene polymorphisms associated with susceptibility to rheumatoid arthritis disease and its severity in Brazilian patients. Tissue Antigens.

[32] Mariaselvam CM, Chaaben AB, Salah S, Charron D, Krishnamoorthy R, Tamouza R (2015). Human leukocyte antigen-G polymorphism influences the age of onset and autoantibody status in rheumatoid arthritis. Tissue Antigens.

[33] Silva HPV, Ururahy MAG, Souza KSC, Loureiro MB, Oliveira YMC, Oliveira GHM (2016). The association between the HLA-G 14-bp insertion/deletion polymorphism and type 1 diabetes. Genes Immun.

[34] Lin A, Yan WH, Xu HH, Tang LJ, Chen XF, Zhu M (2007). 14 bp deletion polymorphism in the HLA-G gene is a risk factor for idiopathic dilated cardiomyopathy in a Chinese Han population. Tissue Antigens.

[35] Veit TD, Vianna P, Scheibel I, Brenol CV, Brenol JC, Xavier RM (2008). Association of the HLA-G 14-bp insertion/deletion polymorphism with juvenile idiopathic arthritis and rheumatoid arthritis. Tissue Antigens.

[36] Rizzo R, Hviid TV, Govoni M, Padovan M, Rubini M, Melchiorri L (2008). HLA-G genotype and HLA-G expression in systemic lupus erythematosus: HLA-G as a putative susceptibility gene in systemic lupus erythematosus. Tissue Antigens.

[37] Veit TD, Cordero EA, Mucenic T, Monticielo OA, Brenol JC, Xavier RM (2009). Association of the HLA-G 14 bp polymorphism with systemic lupus erythematosus. Lupus.

[38] Wu FX, Wu LJ, Luo XY, Tang Z, Yang MH, Xie CM (2009). Lack of association between HLA-G 14-bp polymorphism and systemic lupus erythematosus in a Han Chinese population. Lupus.

[39] Martelli-Palomino G, Pancotto JA, Muniz YC, Mendes-Junior CT, Castelli EC, Massaro JD (2013). Polymorphic sites at the 3’ untranslated region of the HLA-G gene are associated with differential hla-g soluble levels in the Brazilian and French population. PLoS One.

[40] Castelli EC, Mendes-Junior CT, Deghaide NH, de Albuquerque RS, Muniz YC, Simoes RT (2010). The genetic structure of 3’untranslated region of the HLA-G gene: polymorphisms and haplotypes. Genes Immun.

[41] Castelli EC, Ramalho J, Porto IO, Lima TH, Felicio LP, Sabbagh A (2014). Insights into HLA-G genetics provided by worldwide haplotype diversity. Front Immunol.

[42] Rizzo R, Farina I, Bortolotti D, Galuppi E, Rotola A, Melchiorri L (2013). HLA-G may predict the disease course in patients with early rheumatoid arthritis. Hum Immunol.

[43] Verbruggen LA, Rebmann V, Demanet C, De Cock S, Grosse-Wilde H (2006). Soluble HLA-G in rheumatoid arthritis. Hum Immunol.

[44] Rizzo R, Rubini M, Govoni M, Padovan M, Melchiorri L, Stignani M (2006). HLA-G 14-bp polymorphism regulates the methotrexate response in rheumatoid arthritis. Pharmacogenet Genomics.

[45] Kooloos WM, Wessels JA, van der Straaten T, Allaart CF, Huizinga TW, Guchelaar HJ (2010). Functional polymorphisms and methotrexate treatment outcome in recent-onset rheumatoid arthritis. Pharmacogenomics.

[46] Baricordi OR, Govoni M, Rizzo R, Trotta F (2007). In rheumatoid arthritis, a polymorphism in the HLA-G gene concurs in the clinical response to methotrexate treatment. Ann Rheum Dis.

[47] Stamp LK, O’Donnell JL, Chapman PT, Barclay ML, Kennedy MA, Frampton CM (2009). Lack of association between HLA-G 14 bp insertion/deletion polymorphism and response to long-term therapy with methotrexate response in rheumatoid arthritis. Ann Rheum Dis.

[48] Lee YH, Bae SC, Song GG (2015). Meta-analysis of associations between functional HLA-G polymorphisms and susceptibility to systemic lupus erythematosus and rheumatoid arthritis. Rheumatol Int.

[49] Kim SK, Jeong KH, Kang IJ, Chung JH, Shin MK, Lee MH (2015). Relationship between the HLA-G 14bp insertion/deletion polymorphism and susceptibility to autoimmune disease: a meta-analysis. Genet Mol Res.

