# Supplementary figures and images for: “HLA-G 3′UTR gene polymorphisms and rheumatic heart disease: a familial study among South Indian population”
Source: Pediatr Rheumatol Online J. 2017 Feb 1;15:10. doi: 10.1186/s12969-017-0140-x (PMC5286793; doi:10.1186/s12969-017-0140-x)

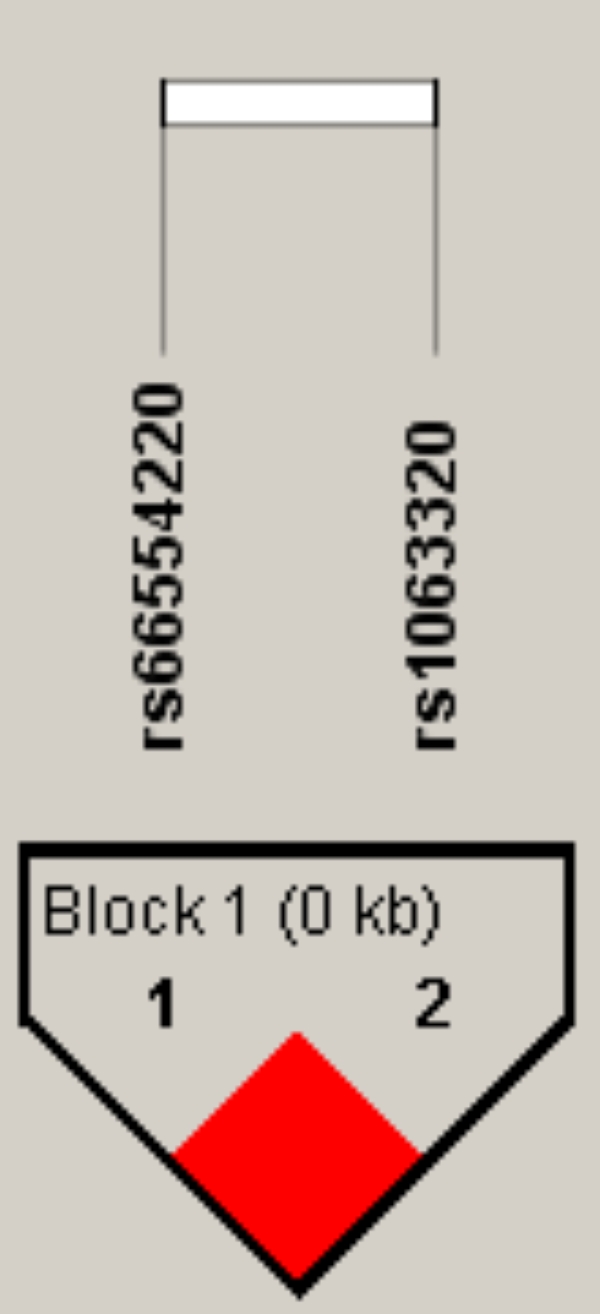

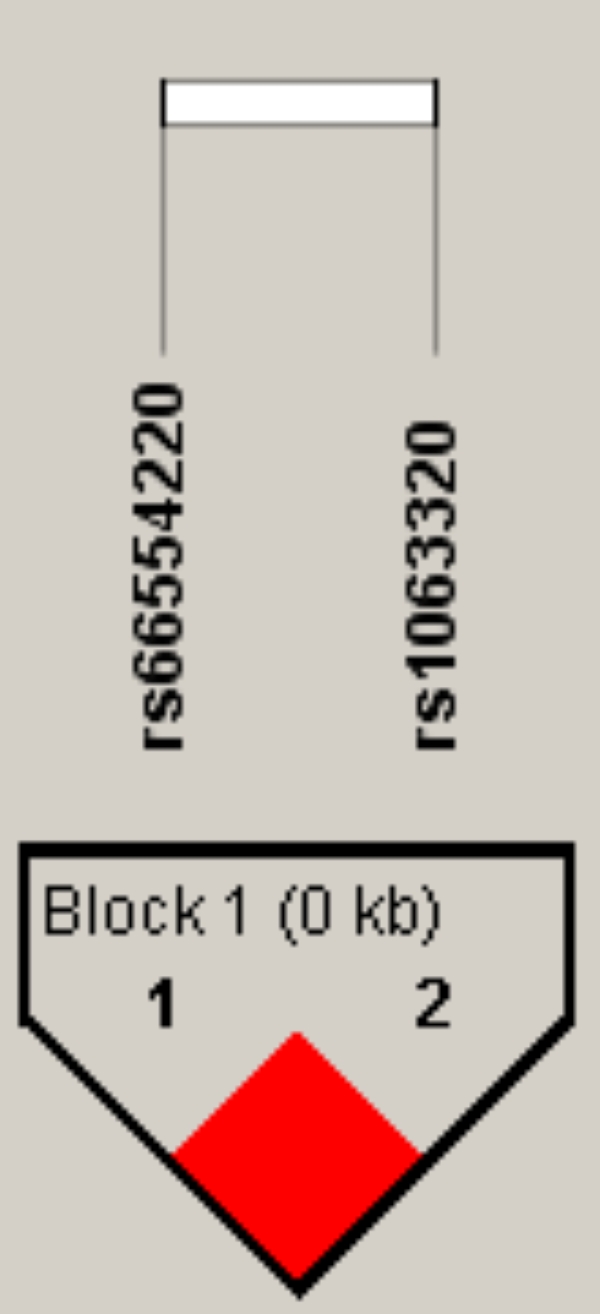

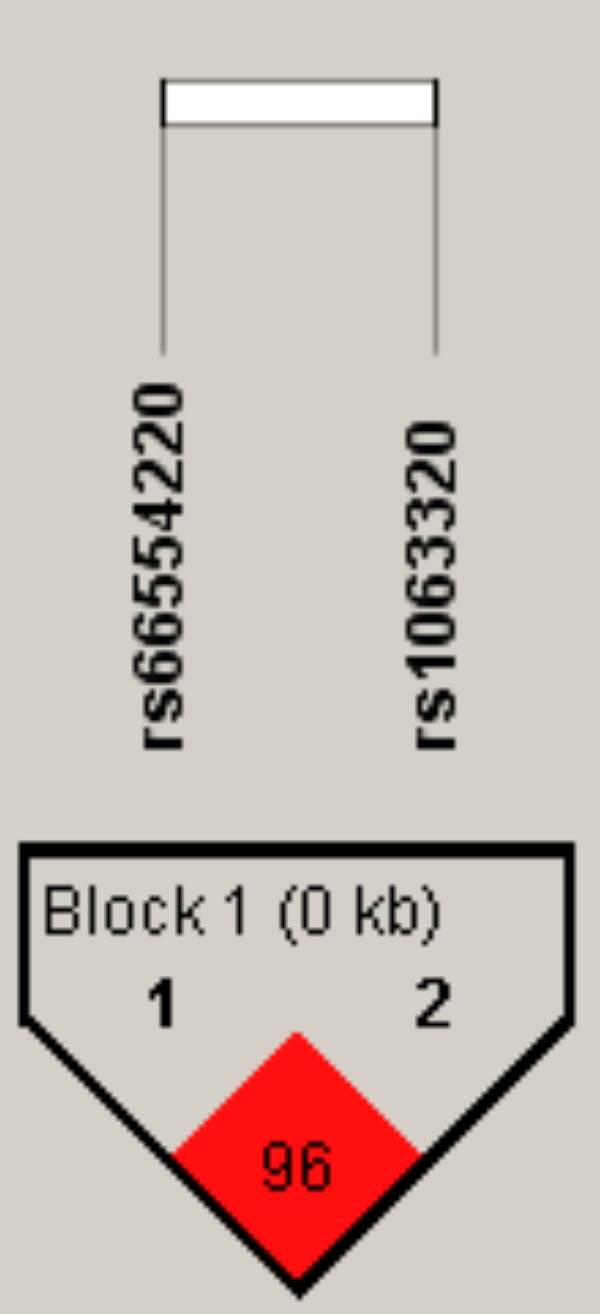


A B C

Supplement: Additional file 1: Figure S1. — Pairwise linkage disequilibrium based on 2 HLA-G Polymorphisms using HaploView 4.2. Red squares represent high pair-wise linkage disequilibrium. The numbers in the individual square are D' multiplied by 100. A. Healthy siblings B. RHD patients C. Trio families. (DOC 301 kb) [file 12969_2017_140_MOESM1_ESM.doc]
